# Scale-up interventions—Moving from pilot projects to larger implementation settings

**DOI:** 10.1186/s12978-024-01843-9

**Published:** 2024-07-12

**Authors:** Kathya Lorena Cordova-Pozo, Jose M. Belizán

**Affiliations:** 1 Institute for Management Research, Radboud University, Nijmegen, The Netherlands; 2 Institute for Clinical Effectiveness and Health Policy (IECS), Buenos Aires, Argentina

This year, we commemorate the 30th anniversary of the International Conference on Population and Development and its landmark Plan of Action that included adolescent sexual and reproductive health in the agendas of most countries [1]. Similar commitments were also prompting actions to improve adolescent sexual and reproductive health (ASRH), particularly the Millennium Development Goals (MDGs, 2000–2015), then the Sustainable Development Goals (SDGs, 2015–2030), and other specific provisions to address ASRH. Therefore, many governments and NGO-led projects included adolescent health in its policies and strategies to address them, which increased data and evidence on what works and what does not. According to Chandra-Mouli, the discourse has evolved from asking “*Why do we need to address adolescents?* through: *What do we need to do to address adolescents?* And then to: *How do we do what needs to be done to address adolescence in our particular social, cultural, and economic context?”* over the last three decades [2]*.*

In the past years, many interventions have been carried out around the world. All types of interventions, being biomedical, educational, social, or economic were implemented to improve promotion, prevention, treatment or rehabilitation, and support for adolescents. Firstly, with the evidence at hand, the Guttmacher-Lancet Commission Report recommends interventions that work [3]. Secondly, there are some examples of how some countries successfully scaled-up comprehensive sexuality education like Uruguay, Mexico, Nigeria, Senegal, Pakistan, and India [4], and successful adolescent pregnancy programs, like Argentina, Ethiopia, Moldova, Thailand [5]. Thirdly, we can draw some lessons from this and from the experience of Chandra-Mouli while implementing the interventions (a) evidence-base interventions of what works and what not, (b) effective intervention-delivery mechanisms to ensure fidelity, (c) sustainable mechanisms that ensure feasibility and acceptability to those being targeted in the local context, and (d) scale up mechanisms with real accountability for the execution and the results of the implemented programs, creating support within the civil society and other stakeholders [4].

The authors consider these very important characteristics, (a) it is important to increase monitoring within similar indicators for wider comparability. This will improve the delivery-mechanisms and effectiveness (b). Increase funding in macro-fiscal analysis and policy modelling to further suggest health programs that are feasible and sustainable for each context (c), and while demonstrating health service response, this can further increase legitimacy and accountability for the health system and its governments (d). While these characteristics are important to carry scale-up interventions, factors like financial constraints, human resource shortages, and logistic problems -e.g., medicines and supplies; hinder their ability to deliver the interventions as planned. In addition, a list of what works, ASRH improvements, and the challenges faced during implementation are also illustrated by the papers in this supplement [4, 6–12].

## Key suggestions

This supplement contains an interesting set of papers that transmit the following messages about what works, ASRH improvements, and the challenges faced during implementation.

- Adolescent pregnancy and interventions. A scoping review in 28 articles show-casing Nigeria indicate the three most contributing factors are access to CSE, wealth or ethnicity, and early marriage practices and the most advised interventions are the CSE, and parental support. Still, caution needs to be taken as 21 research studies are based on different types of surveys, 3 have DHS data, and 4 are qualitative data [11]. A similar study in India also highlighted the access to CSE, the lack of access to contraception, and the necessity of abortion practices [12]. A cluster randomized trial tested the effectiveness of CSE in Odisha, India, evidencing an improved knowledge and attitude in pregnancy, contraception, and STI/HIV/AIDS using the International Technical Guidance on Sexuality Education [9].
- Harmful practices.
  o Child marriage. Overall, it was evidenced that trends are going down in most of the countries. Not all countries have reduced the prevalence rates equally and there is work to do for those that are being left behind. Some factors mentioned for this success are the health system, education, income distribution, law enforcement, economy [8].
  o Female Genital Mutilation: Showed that while 14 countries had a significant declining trend, other 13 did not. These 13 countries show a homogenous distribution of FGM in the population, but the 14 countries have a distribution that affects the poorer or the ones living in rural areas generating inequality between and within countries. This paper shows the economic burden that FGM creates while there is a delay toward the elimination of this practice [6].
- Increase stakeholder participation and policymaking. A framework is suggested for a CSE scale-up trajectory where civil society organizations (CSOs) could make a valuable contribution towards this end. This paper illustrates the experience in Indonesia indicating that it was tested in Benin, India, and Zambia [7]. Other paper shows the use of the value clarification and attitude transformation engagements (VCAT) methodology to engage stakeholders to think about what they should do to improve ASRH [10].
- Data availability. The papers highlight the need for collecting more data and more regularly to measure the progress as well as data for boys [8, 10, 11, 13], and the alignment of methods for designing interventions [11]. This will help long-term analysis and comparative evaluation of progress, which is in line with other authors [8, 14].
- Technical Assistance Coordination Mechanism. The WHO created a framework to support programmes that aim to improve ASRH in a timely, efficient, effective way for strengthening capacity [4].

These manuscripts vision the future within a combination of social movements and strong health governance to scale up programs to improve the sexual and reproductive health of adolescents. It is also important to acknowledge that there is a big path advanced on this topic because of Venkatraman Chandra-Mouli who has worked 30 years at the World Health Organization (WHO), focusing on adolescent sexual and reproductive health (ASRH), which includes the UNDP/UNFPA/ UNICEF/WHO/World Bank Human Reproductive Programme. His work has extensively contributed to the scientific evidence of the topic, supported the implementation of evidence to action, and has stimulated and supported research studies or evaluations, and contributed to strengthening policies and program in different social, cultural, and economic contexts. His numerous publications include scientific publications, evidence reviews, policy and programmatic guidelines, advocacy documents, training and self-learning materials and research/evaluation tools.

Evidence shows that there is scale-up going on and there are lessons to draw from those experiences, and other countries could learn from those. Effective implementation and monitoring of evidence-base interventions, adequate investment for scale-up, stakeholder engagement with a focus on quality and equity is needed to enable adolescents to grow and develop in good health.
